# Effects of magnesium sulfate on periarticular infiltration analgesia in total knee arthroplasty: a prospective, double-blind, randomized controlled trial

**DOI:** 10.1186/s13018-023-03790-w

**Published:** 2023-04-14

**Authors:** Chengcheng Zhao, Liying Wang, Liyile Chen, Qiuru Wang, Pengde Kang

**Affiliations:** 1 Department of Orthopaedics, Orthopaedic Research Institute, West China Hospital, Sichuan University, No. 37 Guoxue Road, Chengdu, 610041 Sichuan China; 2 Department of Operating Room, West China Hospital, Sichuan University, Chengdu, China; 3 West China School of Nursing, Sichuan University, Chengdu, China

**Keywords:** Total knee arthroplasty, Periarticular infiltration analgesia, Pain, Cocktail, Magnesium sulfate

## Abstract

**Background:**

To investigate whether adding magnesium sulfate to a periarticular infiltration analgesia (PIA) cocktail could improve pain control and functional outcomes in patients undergoing total knee arthroplasty (TKA).

**Methods:**

Ninety patients were randomly assigned to the magnesium sulfate and control groups, with 45 patients in each group. In the magnesium sulfate group, patients were given a periarticular infusion of a cocktail of analgesics consisting of epinephrine, ropivacaine, magnesium sulfate, and dexamethasone. The control group received no magnesium sulfate. The primary outcomes consisted of visual analogue scale (VAS) pain scores, postoperative morphine hydrochloride consumption for rescue analgesia, and time to first rescue analgesia. Secondary outcomes were postoperative inflammatory biomarkers (IL-6 and CRP), postoperative length of stay, and knee functional recovery (assessed by knee range of motion, quadriceps strength, daily mobilization distance, and time to first straight-leg raising). Tertiary outcomes included the postoperative swelling ratio and complication rates.

**Results:**

Within 24 h of surgery, patients in the magnesium sulfate group had markedly lower VAS pain scores during motion and at rest. After the addition of magnesium sulfate, the analgesic effect was dramatically prolonged, leading to a reduction in morphine dosage within 24 h and the total morphine dosage postoperatively. Postoperative inflammatory biomarker levels were significantly reduced in the magnesium sulfate group compared with the control. There were no considerable differences between the groups in terms of the postoperative length of stay and knee functional recovery. Both groups had similar postoperative swelling ratios and incidences of complications.

**Conclusions:**

The addition of magnesium sulfate to the analgesic cocktail for PIA can prolong postoperative analgesia, decrease the consumption of opioids, and effectively alleviate early postoperative pain after TKA.

*Trial registration*: Chinese Clinical Trial Registry, ChiCTR2200056549. Registered on 7 February 2022, https://www.chictr.org.cn/showproj.aspx?proj=151489.

## Background

Total knee arthroplasty (TKA) is the most effective way to alleviate knee pain and enhance knee function in patients with end-stage degenerative knee disease [1]. However, previous investigations have shown that over 60% of patients who undergo TKA experience moderate-to-severe postoperative pain [2, 3]. Postoperative pain is a major factor hindering enhanced recovery in patients [4]. Multimodal pain management regimens after TKA can decrease pain scores, reduce the length of stay, promote faster recovery, and improve patient satisfaction [4–6]. Periarticular infiltration analgesia (PIA) is a pivotal technique in multimodal TKA pain management [2, 3, 7, 8]. It can deliver desirable pain relief and assist in the maintenance of muscle strength in the absence of complications associated with opioids [9]. The commonly used medications in LIA were mixtures of local anesthetics and other adjuvants, which enhance the analgesic effect. The prepared mixed medications were usually referred to as "cocktails" by researchers. Nevertheless, there is no gold standard regarding the quantity together with the composition of drugs available for analgesic cocktails [9, 10].

Although numerous adjuvants, such as epinephrine, morphine, clonidine, non-steroidal anti-inflammatory drugs (NSAIDs), and corticosteroids, have been added to local anesthetics to improve their analgesic effects [2, 3, 11–13], the duration of postoperative analgesia remains a limiting factor. To date, no adjuvant has been shown to significantly prolong the duration of local anesthetics used in PIA. Magnesium sulfate is a potent postoperative analgesic adjunct [14]. Its analgesic ability seems to be related to the antagonism of *N*-methyl-d-aspartate (NMDA) receptors in the peripheral and central nervous systems or modulation of calcium influx into cells [15, 16]. Magnesium sulfate has been assessed as an adjunct to ropivacaine-induced brachial plexus nerve block and was found to improve ropivacaine action time [17]. Furthermore, magnesium sulfate has anti-inflammatory properties. In an inflammatory state, pain can be induced by peripheral or central sensitization [16].

Few clinical studies have documented the use of magnesium sulfate for the treatment of PIA after TKA. In current clinical practice, epinephrine and glucocorticoid are commonly used as adjuncts to improve the analgesic efficacy of local anesthetics [9, 18]. This study examined the efficacy of magnesium sulfate as a component of an analgesic cocktail used in PIA for patients undergoing TKA. Based on a traditional cocktail consisting of ropivacaine, epinephrine, and dexamethasone, we hypothesized that the addition of magnesium sulfate to a conventional cocktail would extend the duration of analgesia, improve the relief of early postoperative pain, and decrease inflammation.

## Methods

### Ethics statements

This study was approved by our Clinical Trials and Biomedical Ethics Committee (number: 2021-1232) and registered with the Chinese Clinical Trials Registry (ChiCTR2200056549) on 7 February 2022. Written informed consent was obtained from all patients.

### Study design and patient recruitment

The current study was a randomized, controlled, double-blind, prospective trial. Patients with osteoarthritis who underwent primary unilateral TKA between February 2022 and December 2022 at our institution were included in the present study. The inclusion criteria were as follows: (1) age ranging from 40 to 85 years, (2) American Society of Anesthesiologists (ASA) functional status of I–III, and (3) body mass index (BMI) of 18–36 kg/m^2^. The exclusion criteria were as follows: (1) knee deformity of flexion ≥ 30° or varus-valgus ≥ 30°, (2) hypersensitivity to medications used in this study, (3) chronic opioid use, (4) history of knee surgery (open and arthroscopic surgery), (5) recognized neuromuscular disease, (6) knee infection, and (7) history of cognitive impairment, narcotic dependence, or psychiatric disease.

### Randomization

A list of computer-generated random numbers (Excel; Microsoft Corporation, Redmond, WA, USA) was used to divide patients into two groups. Subsequently, such random numbers were sealed in opaque envelopes by Investigator A, who was blinded to the group allocation and study design. Investigator A asked each patient to choose an envelope on the morning of the operation. The patients were assigned by Investigator B to the magnesium sulfate or control group, following the number in the chosen envelope. Before surgery, Investigator B ensured that anesthesiologists who did not participate in the study prepared the appropriate analgesic cocktail. The surgeon and outcome assessor (Investigator C) were blinded to the treatment group. Another investigator (Investigator D), who was also blinded to the group assignment, performed statistical analysis. By the end of the test (3 months after the operation), the patients were informed of the group to which they belonged.

### Data collection

At admission, the following patient characteristics were recorded: sex, age, BMI, weight, height, visual analogue scale (VAS) pain score for daily activities, side of surgery, knee range of motion (ROM), ASA functional status, quadriceps strength, and time of surgery.

### Perioperative analgesia and management

All TKA procedures were performed by the same surgeon at our hospital. After general anesthesia was administered to the patient, the surgeon made a midline skin incision through the medial parapatellar approach and implanted a cemented prosthesis (DePuy Synthes, New Brunswick, NJ, USA). Before surgery, celecoxib (200 mg) was administered twice daily as a prophylactic analgesic. All patients received 1 g of tranexamic acid intravenously 30 min before skin incision and 3 and 6 h postoperatively. No tourniquets or drainage tubes were used.

PIA was performed by the surgeon. The magnesium sulfate group received 0.2% ropivacaine, magnesium sulfate (2.5 mg/mL), dexamethasone (0.1 mg/mL), or epinephrine (2.0 μg/mL). The control group received 0.2% ropivacaine, dexamethasone (0.1 mg/mL), and epinephrine (2.0 μg/mL). The volume of the analgesic cocktail was 100 mL in both groups. All methods used in PIA were the same for both groups, except for the analgesic cocktail composition. Before implantation of the prosthesis, the cocktail (20 mL) was injected into the joint capsule posteriorly, and the cocktail (20 mL) served as an infiltrating analgesic for the lateral and medial collateral ligaments. After implantation, the quadricep and retinacular tissues were infiltrated with the cocktail (20 mL), and the subcutaneous tissues and fat were infiltrated with the cocktail (40 mL). Systemic corticosteroids were not administered.

All patients were given an ice compression on their return to the ward; however, no pain pumps were used. Oral celecoxib (200 mg) was administered twice daily to control postoperative pain. If patients experienced intolerable pain, additional morphine hydrochloride (5 or 10 mg) was subcutaneously injected as rescue analgesia. To prevent venous thromboembolism (VTE), 0.2 mL of enoxaparin was administered 12 h postoperatively, with subsequent dose increases (0.4 mL per day) before discharge. After discharge, 10 mg of rivaroxaban was administered once a day for 2 weeks.

### Outcomes and follow-up

The primary outcomes included the time to the first rescue analgesia, morphine consumption, and pain score. During motion (knee flexion of 45°) and at rest, postoperative pain was scored on a VAS scale (0–10, 0 represents no pain, whereas 10 represents the most pain) [19].

Secondary outcomes included inflammatory biomarkers (interleukin [IL]-6 and C-reactive protein [CRP]) and recovery of knee function as determined by quadriceps strength, daily mobilization distance, ROM, and time to first straight-leg raising. Fasting blood samples were collected on the morning of postoperative days 1, 2, and 3 to determine CRP and IL-6 levels. A protractor was used to measure ROM three times a day, 6 h apart, and the optimal value was used as the value for that day. Quadriceps strength was determined by asking the patient to flex the knees and hips, and was evaluated on a scale of 0–5, with 0 representing the worst strength and 5 representing the most optimal strength. For the daily distance of mobilization, patients needed to walk as far as possible in a single attempt, and the distance acquired was recorded. The postoperative length of stay was also recorded.

Tertiary outcomes included adverse reactions, such as wound complications, nausea, vomiting, chronic pain, nerve damage, postoperative infection, VTE, a significant decrease in quadriceps strength, and falls after surgery. Additionally, the knee swelling ratios were compared between the groups. Based on previous research, the swelling ratio was determined as the average postoperative circumference of the inferior and superior patellar poles divided by the preoperative value [20].

To evaluate the aforementioned clinical results, we encouraged all patients to remove their sutures at 3 weeks postoperatively and to return to the hospital for a 3-month follow-up.

### Statistical analysis

The sample size was based on a pilot study involving 30 patients who were excluded from the main study. In the pilot study, morphine consumptions (mean ± standard deviation [SD]) in the magnesium sulfate and control groups on postoperative day 1 were 6.7 ± 5.8 mg and 11.6 ± 7.5 mg, respectively. We computed the smallest sample size of 39 patients in both groups with a bilateral alpha level of 0.05 and power of 90%. Considering the risk of dropout, 45 patients were included in each group.

The Shapiro–Wilk test was used to evaluate data normality. Continuous and categorical data are expressed as mean ± SD together with percentage or number, respectively. The Student *t* test was used to determine the significance of normally distributed data between the groups; Mann–Whitney *U* test was used to determine differences in the ordinal and skewed data between the groups; and Fisher exact probability test, continuity correction test, or Pearson chi-square test was used to determine differences in the categorical data between the groups. Survival analysis (log-rank test and Kaplan–Meier approach) was conducted to analyze the time to first rescue analgesia.

All statistical analyses were performed using SPSS version 25 (IBM Corp, Armonk, NY, USA). Differences were considered significant if *p* < 0.05.

## Results

### Study population

**Fig. 1:**
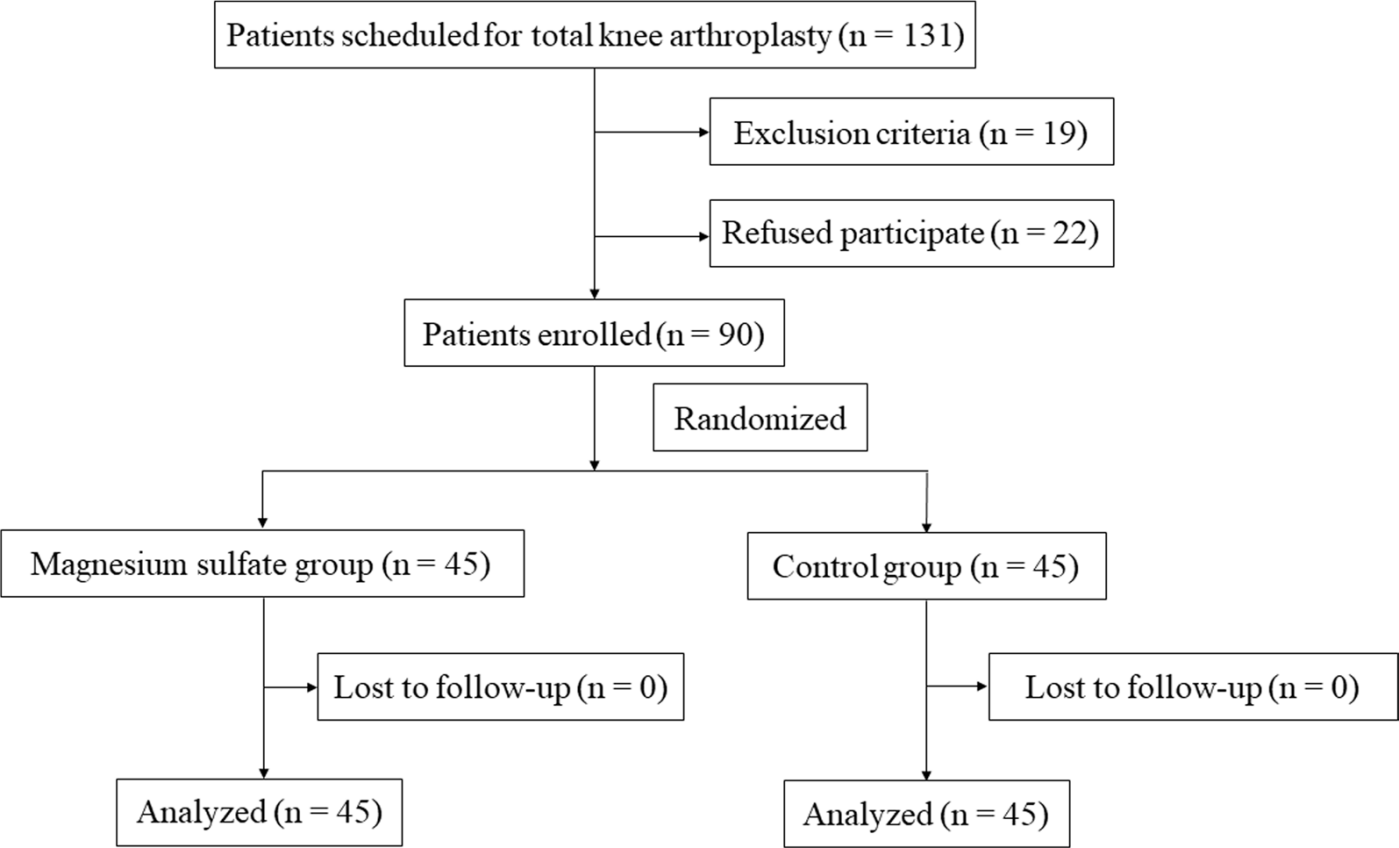
Consolidated Standards of Reporting Trials (CONSORT) flow diagram for the present trial showing patient selection and exclusion

Eligibility was assessed for 131 patients with osteoarthritis; 19 of the patients did not comply with the eligibility criteria, and the remaining 22 were not willing to give their consent. Ultimately, 90 patients were enrolled in this study. No patient withdrew from the study during the postoperative outcome evaluations (Fig. 1). There were no evident differences in preoperative features between the magnesium sulfate and control groups (Table 1).

**Table 1:** Patients’ clinical and demographic characteristics

Characteristic	Control group (*n* = 45)	Magnesium sulfate group (*n* = 45)	*p *value
Age (years)	64.2 ± 8.5	65.9 ± 9.0	0.350^a^
Sex (M/F)	13/32	9/36	0.327^b^
Weight (kg)	61.4 ± 5.3	63.9 ± 10.1	0.147^a^
Height (cm)	155.6 ± 6.1	155.7 ± 7.4	0.968^c^
Body mass index (kg/m^2^)	25.5 ± 3.0	26.4 ± 3.8	0.249^a^
Side of surgery (right/left)	27/18	22/23	0.290^b^
VAS pain score (prior to surgery)	4.47 ± 0.84	4.27 ± 0.75	0.304^c^
Knee ROM (prior to surgery)	111.1 ± 13.8	114.4 ± 12.6	0.211^c^
Quadriceps strength	4.9 ± 0.3	4.9 ± 0.3	0.505^c^
ASA status (I/II/III)	2/35/8	1/30/14	0.127^c^
Time of surgery (min)	64.2 ± 8.3	61.7 ± 7.0	0.132^a^

Values are presented as mean ± standard deviation or number of cases

*M* male, *F* female, *VAS* visual analogue scale, *ROM* range of motion, *ASA* American Society of Anesthesiologists

^a^Student *t* test

^b^Pearson chi-square test

^c^Mann–Whitney *U* test

### Primary outcome

**Fig. 2:**
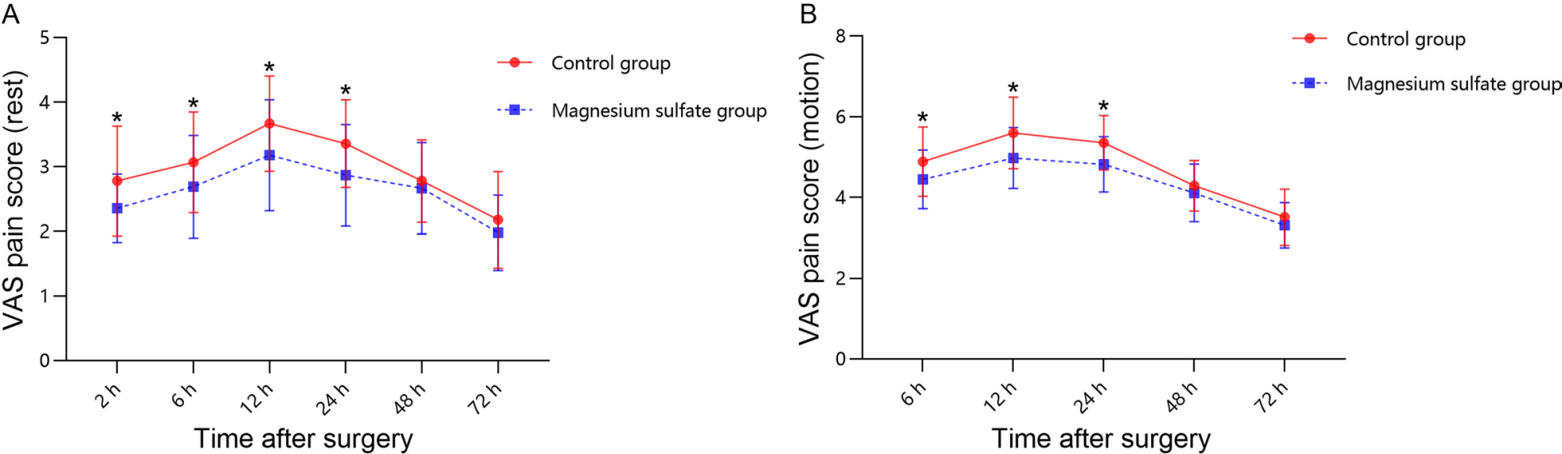
Postoperative VAS pain scores of the two groups presented as mean and standard deviation. **A** Pain scores at rest. **B** Pain scores during motion. * indicates a statistical difference (*p* < 0.05) between the two groups. *VAS* visual analogue scale

**Fig. 3:**
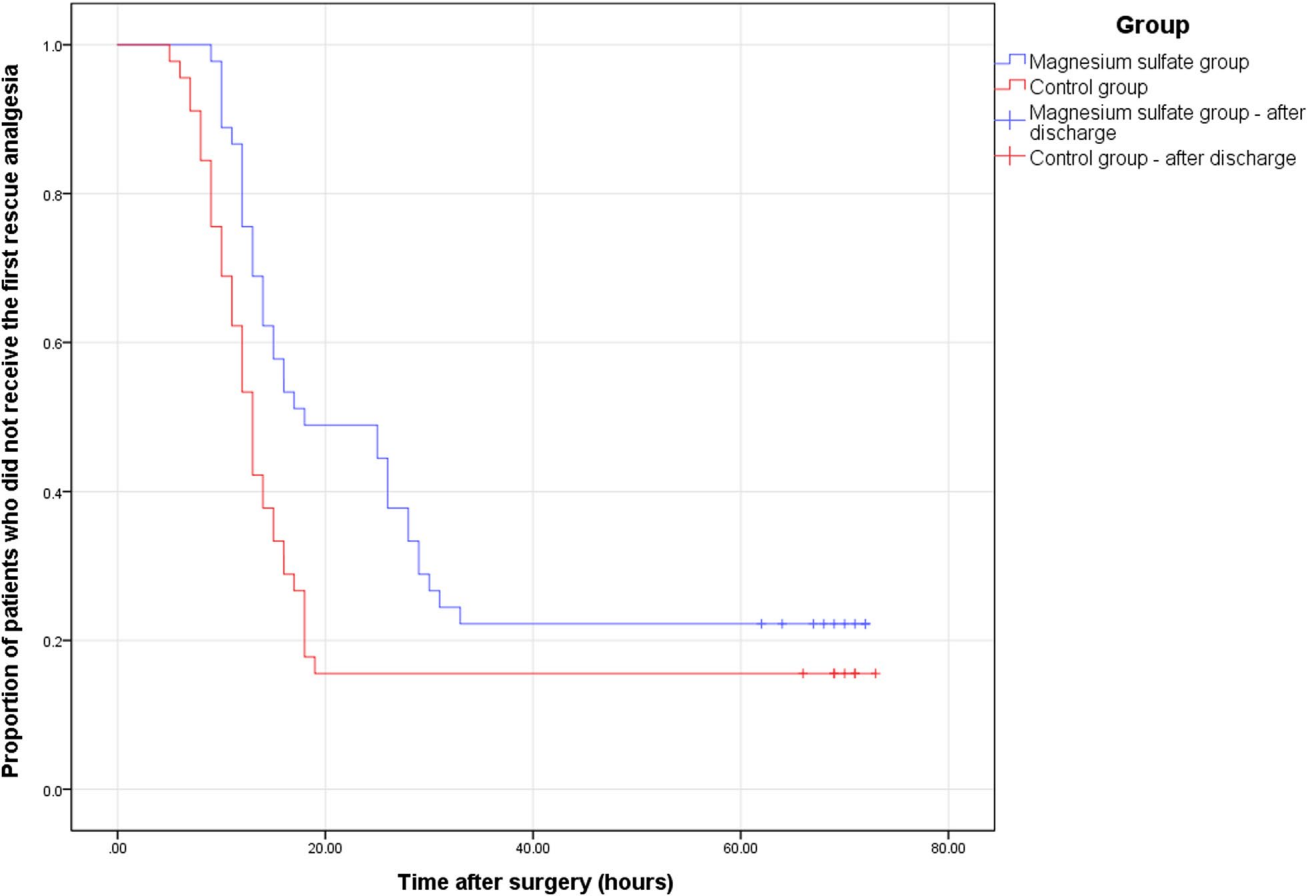
Survival analysis of the time to first rescue analgesia. The difference between the groups is statistically significant (*p* = 0.020, Kaplan–Meier method with log-rank test)

**Fig. 4:**
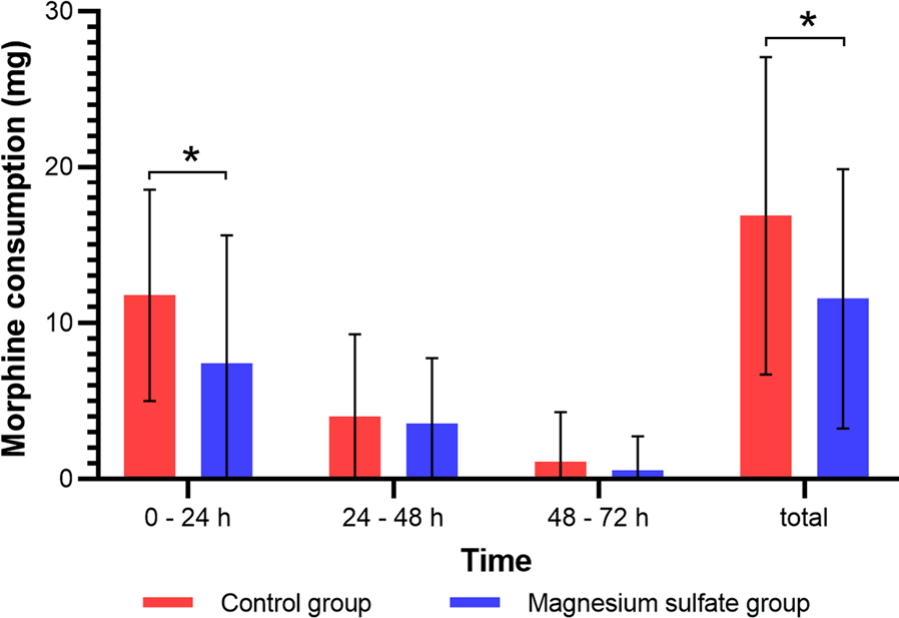
Consumption of morphine hydrochloride postoperatively is presented as mean and standard deviation. * indicates a statistical difference (*p* < 0.05) between the two groups

The VAS scores at rest (Table 2, Fig. 2A) and during motion (Table 2, Fig. 2B) within 24 h after surgery were markedly lower in the magnesium sulfate group than in the control group. In addition, 10 patients (22.22%) in the magnesium sulfate group did not receive rescue analgesia compared with 7 patients (15.56%) in the control group (*p* = 0.419). The magnesium sulfate group showed a considerably longer time to the first rescue analgesia than the control group (18.11 ± 7.64 *vs* 12.08 ± 3.73 h, *p* = 0.020) (Table 2, Fig. 3). The magnesium sulfate group consumed significantly less morphine in the first 24 h postoperatively and consumed less morphine overall (Table 2, Fig. 4). Nevertheless, there was no remarkable difference between the two groups in morphine consumption on days 2 and 3.

**Table 2:** Postoperative pain assessment

Outcome	Control group (*n* = 45)	Magnesium sulfate group (*n* = 45)	*p *value
VAS pain score (rest)^a^			
2 h	2.78 ± 0.85	2.34 ± 0.53	0.020
6 h	3.07 ± 0.78	2.69 ± 0.79	0.013
12 h	3.67 ± 0.74	3.18 ± 0.86	0.008
24 h	3.36 ± 0.68	2.87 ± 0.79	0.002
48 h	2.78 ± 0.64	2.67 ± 0.71	0.351
72 h^†^	2.18 ± 0.75	1.98 ± 0.58	0.205
VAS pain score (motion)^a^			
6 h	4.89 ± 0.86	4.44 ± 0.72	0.017
12 h	5.60 ± 0.89	4.98 ± 0.75	0.001
24 h	5.36 ± 0.68	4.82 ± 0.68	< 0.001
48 h	4.29 ± 0.63	4.11 ± 0.71	0.242
72 h^†^	3.51 ± 0.69	3.31 ± 0.56	0.165
Morphine consumption (mg)^a^			
Within 24 h	11.78 ± 6.76	7.44 ± 8.16	0.008
24–48 h	4.00 ± 5.29	3.56 ± 4.21	0.949
48–72 h	1.11 ± 3.18	0.56 ± 2.19	0.437
Total	16.89 ± 10.19	11.56 ± 8.31	0.012
Time to first rescue analgesia (h)^‡^	12.08 ± 3.73	18.11 ± 7.64	0.020^b^
No morphine cases (*n*, %)	7 (15.56%)	10 (22.22%)	0.419^c^

Values are presented as mean ± standard or *n* (%)

*VAS*, visual analogue scale

^a^Mann–Whitney *U* test

^b^Kaplan–Meier method with log-rank test

^c^Pearson chi-square test

^†^If the patient’s hospital stay was < 72 h, the pain score at discharge was recorded instead of at 72 h after surgery

^‡^Patients who did not receive rescue analgesia were excluded

### Secondary outcomes

**Fig. 5:**
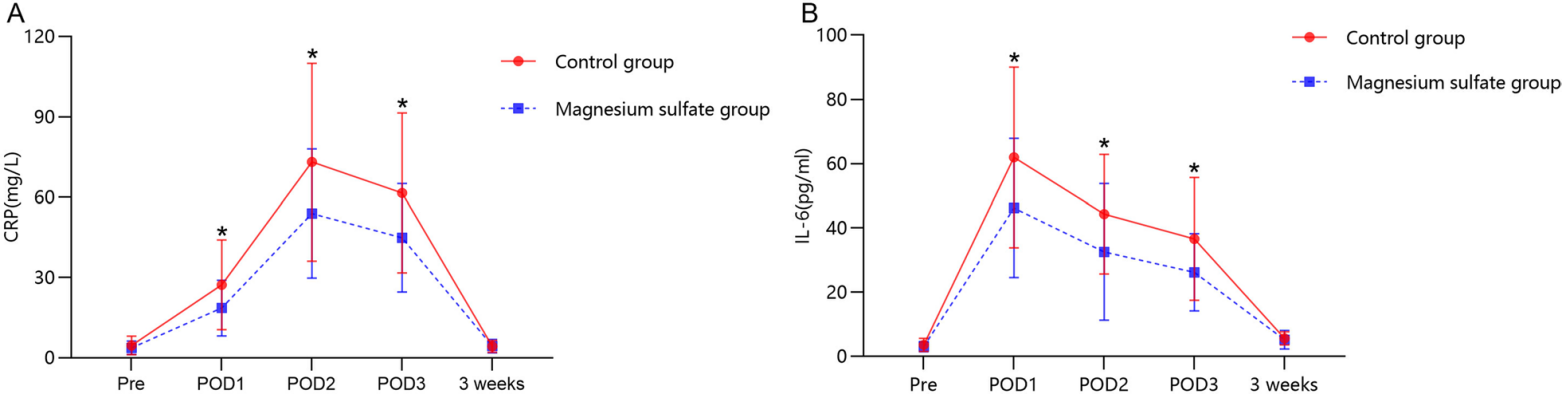
Pre- and postoperative levels of the inflammatory biomarkers presented as mean and standard deviation. **A** CRP and **B** IL-6. * indicates a statistical difference (*p* < 0.05) between the two groups.* POD* postoperative day, *CRP* C-reactive protein, *IL-6* interleukin-6

IL-6 and CRP levels were remarkably lower in the magnesium sulfate group than in the control group on postoperative days 1, 2, and 3 (Fig. 5A, B). Regarding postoperative functional recovery, there were no apparent differences between the groups in terms of the postoperative daily mobilization distance, quadriceps strength, and knee ROM (Table 3). There were no marked differences between the groups in terms of the postoperative length of stay and time to first straight-leg raising (Table 3).

**Table 3:** Postoperative functional recovery

Outcome	Control group (*n* = 45)	Magnesium sulfate group (*n* = 45)	*p *value^a^
Knee ROM (°)			
Postoperative day 1	87.0 ± 9.0	89.0 ± 6.3	0.465
Postoperative day 2	95.3 ± 7.3	95.8 ± 5.1	0.834
Postoperative day 3	104.7 ± 7.2	106.1 ± 5.0	0.258
3 months	115.7 ± 6.2	117.9 ± 4.8	0.099
Quadriceps strength			
Postoperative day 1	3.6 ± 0.7	3.7 ± 0.9	0.727
Postoperative day 2	4.2 ± 0.5	4.4 ± 0.6	0.120
Postoperative day 3	4.7 ± 0.5	4.8 ± 0.4	0.635
3 months	4.9 ± 0.3	5.0 ± 0.2	0.647
Daily mobilization distance (m)			
Postoperative day 1	11.0 ± 5.6	12.4 ± 3.9	0.090
Postoperative day 2	18.7 ± 6.5	19.2 ± 6.4	0.646
Postoperative day 3	30.6 ± 7.3	31.6 ± 6.1	0.448
Time to first straight-leg raising (h)	10.9 ± 5.4	11.4 ± 5.9	0.887
Postoperative length of stay (h)	69.4 ± 3.0	68.4 ± 3.2	0.160

Values are presented as mean ± standard deviation

*ROM*, range of motion

^a^Mann–Whitney *U* test

### Tertiary outcomes

There was no remarkable difference in the postoperative swelling ratios between the groups (*p* > 0.05, Table 4). The incidences of vomiting (*p* = 0.270), nausea (*p* = 0.455), chronic pain (*p* = 0.266), and wound complications (*p* = 0.711) were similar in both groups during postoperative hospitalization (Table 4). Over the 3-month follow-up period, there was no nerve damage, postoperative infection, VTE, significant decrease in quadriceps strength, fall after surgery, mortality, or readmission.

**Table 4:** Postoperative swelling ratio and complications

Outcome	Control group (*n* = 45)	Magnesium sulfate group (*n* = 45)	*p *value
Swelling ratio (%)			
Postoperative day 1	103.2 ± 1.6	103.4 ± 2.1	0.625^a^
Postoperative day 2	105.1 ± 2.1	104.9 ± 2.1	0.631^a^
Postoperative day 3	105.1 ± 2.3	104.3 ± 1.8	0.058^a^
Postoperative complications			
Nausea	12 (26.7%)	9 (20.0%)	0.455^b^
Vomiting	10 (22.2%)	6 (13.3%)	0.270^b^
Wound complications	5 (11.1%)	3 (6.7%)	0.711^c^
Chronic pain	6 (13.3%)	2 (4.4%)	0.266^c^
Nerve damage	0 (0)	0 (0)	
Postoperative infection	0 (0)	0 (0)	
Venous thrombotic events	0 (0)	0 (0)	
Significant decrease of quadriceps strength	0 (0)	0 (0)	
Fall after surgery	0 (0)	0 (0)	
3-month mortality	0 (0)	0 (0)	
3-month readmission	0 (0)	0 (0)	

Values are presented as mean ± standard deviation or *n* (%)

^a^Student *t* test

^b^Pearson chi-square test

^c^Continuity correction test

## Discussion

The current study aimed to identify whether the utilization of magnesium sulfate in PIA could enhance the effectiveness of postoperative analgesia compared with the traditional cocktail after TKA. We found that the addition of magnesium sulfate could enhance postoperative early pain relief, provide a longer duration of analgesia, reduce morphine consumption, and decrease blood inflammatory biomarkers. Nevertheless, other outcome measurements, including postoperative functional recovery, length of stay, swelling ratio, and occurrence of complications, were similar between the groups.

TKA features osteotomy, soft tissue release, and deep surgical sites and can lead to exposure of the sensory nerve fibers located around the patella with insufficient protection of the soft tissues, forcing patients to endure severe postoperative pain [1, 21, 22]. The use of perioperative analgesia is of great significance for patients undergoing TKA to return to exercise and enhance recovery [2]. PIA is an effective intraoperative intervention to relieve pain [2, 9, 23], as it provides a desirable analgesic effect and assists in maintaining muscle strength [3, 10]. However, the duration of postoperative analgesia is always a limiting factor in PIA. Therefore, researchers have attempted to add various adjuvants to local anesthetics, including sympathetic nervous system modulators, opioids, NSAIDs, and corticosteroids, to extend the duration of their efficacy, but have not achieved satisfactory results [3, 9, 11–13]. This prompted us to try to find new adjuvants, and we chose to add magnesium sulfate to the clinically common cocktail.

Ropivacaine has been demonstrated to be a safer substitute than bupivacaine because of its lower central nervous system and cardiovascular toxicity [24]. Ropivacaine is a long-acting amide local anesthetic that has the benefits of a precise analgesic effect, long duration, and rapid onset. Therefore, ropivacaine was used in the present study. Previous studies have shown that epinephrine and corticosteroids could enhance the analgesic effect of ropivacaine [9, 10, 18, 25, 26]; therefore, we decided to choose a cocktail composed of dexamethasone, epinephrine, and ropivacaine as a control.

Excellent postoperative analgesia while minimizing opioid consumption is a major goal of TKA [27]. In the present study, patients who received the magnesium sulfate cocktail had dramatically lower consumption of morphine 24 h postoperatively and lower total morphine consumption, as well as lower pain scores on the VAS at 24 h postoperatively than the controls. Nevertheless, it is noteworthy that all differences in VAS scores between the groups did not surpass the minimal clinically important difference (MCID) reported [28, 29]. Additionally, the absolute decrease in morphine consumption did not achieve the reported MCID. The magnesium sulfate group showed no improvement in functional recovery after surgery, and the postoperative hospital stay was not reduced. Desirable early functional exercise is essential to minimize joint stiffness, deep vein thrombosis, postoperative infections, and other complications due to inactivity [30, 31]. However, adding magnesium sulfate to the cocktail did not show any advantage in terms of enhanced recovery after surgery. The additional analgesic effect of magnesium sulfate used in the current study was mild. There is still a requirement for prospective researchers to determine the ideal PIA cocktail.

The mechanism of enhanced analgesia by magnesium sulfate may be that magnesium prevents the activation of NMDA receptors [15, 16, 32], which play an essential role in transmitting information about central pain and modulating acute hyperalgesia [33]. NMDA receptors have a high permeability to calcium ions. NMDA receptor activation results in the influx of calcium ions into the cell, which increases the excitability of spinal dorsal horn neurons, results in the development of central sensitization, and lowers the pain threshold after injury [34]. By blocking the activation of dorsal horn NMDA receptors caused by excitatory amino acid transmitters, such as aspartate and glutamate, antagonists can prevent and abolish hypersensitization once built [35].

TKA can result in a local and systemic inflammatory response as well as increased levels of various inflammatory markers. Inflammation is among the major causes of postoperative pain [36, 37]. IL-6 and CRP levels were markedly lower in the magnesium sulfate group than in the control group on postoperative days 1, 2, and 3, demonstrating that the addition of magnesium sulfate to the cocktail contributed to a decrease in inflammation. Magnesium sulfate may have anti-inflammatory properties by antagonizing NMDA receptors, the phosphoinositide 3-kinase/Akt pathway, and inhibiting inflammatory neuromodulators by activating the neuroendocrine pathway [38].

Considering several limitations, our results should be cautiously interpreted. First, the magnesium sulfate doses were selected according to the recommendations of previous studies. Varying drug doses may lead to diverse outcomes, and future investigations are desirable to confirm this. Second, the sample size was based on morphine consumption, but it may have been insufficient power for other outcomes. Third, other multimodal analgesic modalities, such as peripheral nerve blocks, regional anesthesia, and general corticosteroids, were not included, which may have contributed to the outcomes. Furthermore, the study population consisted mostly of women (75.0%). Studies have demonstrated sex-based differences in pain perception [39]. A larger sample size may allow us to investigate the underlying sex differences in functional recovery and sensitivity to postoperative pain.

## Conclusions

The addition of magnesium sulfate to an analgesic cocktail for PIA prolongs postoperative analgesia, decreases the consumption of opioids, and effectively alleviates early postoperative pain after TKA, but does not accelerate functional recovery. However, these findings require further confirmation in future studies.

## Data Availability

The datasets used and/or analyzed during the current study are available from the corresponding author on reasonable request.

## Abbreviations

TKA: Total knee arthroplasty
PIA: Periarticular infiltration analgesia
NSAIDs: Non-steroidal anti-inflammatory drugs
NMDA: *N*-methyl-d-aspartate
ASA: American Society of Anesthesiologists
BMI: Body mass index
VAS: Visual analogue scale
ROM: Range of motion
VTE: Venous thromboembolism
IL: Interleukin
CRP: C-reactive protein
SD: Standard deviation
MCID: Minimal clinically important difference

## References

[1] Li D, Alqwbani M, Wang Q, Liao R, Yang J, Kang P (2020). Efficacy of adductor canal block combined with additional analgesic methods for postoperative analgesia in total knee arthroplasty: a prospective, double-blind, randomized controlled study. J Arthroplasty.

[2] Karam JA, Schwenk ES, Parvizi J (2021). An update on multimodal pain management after total joint arthroplasty. J Bone Jt Surg Am.

[3] Wang Q, Sun J, Hu Y, Zeng Y, Hu J, Yang J, Kang P (2020). Effects of morphine on peri-articular infiltration analgesia in total knee arthroplasty: a prospective, double-blind, randomized controlled trial. Int Orthop.

[4] Yu S, Dundon J, Solovyova O, Bosco J, Iorio R (2018). Can multimodal pain management in TKA eliminate patient-controlled analgesia and femoral nerve blocks?. Clin Orthop Relat Res.

[5] Kopp SL, Børglum J, Buvanendran A, Horlocker TT, Ilfeld BM, Memtsoudis SG, Neal JM, Rawal N, Wegener JT (2017). Anesthesia and analgesia practice pathway options for total knee arthroplasty: an evidence-based review by the american and european societies of regional anesthesia and pain medicine. Reg Anesth Pain Med.

[6] Zhao J, Davis SP (2019). An integrative review of multimodal pain management on patient recovery after total hip and knee arthroplasty. Int J Nurs Stud.

[7] Fenten MGE, Bakker SMK, Scheffer GJ, Wymenga AB, Stienstra R, Heesterbeek PJC (2018). Femoral nerve catheter vs local infiltration for analgesia in fast track total knee arthroplasty: short-term and long-term outcomes. Br J Anaesth.

[8] Kurosaka K, Tsukada S, Seino D, Morooka T, Nakayama H, Yoshiya S (2016). Local infiltration analgesia versus continuous femoral nerve block in pain relief after total knee arthroplasty: a randomized controlled trial. J Arthroplasty.

[9] Pepper AM, Mercuri JJ, Behery OA, Vigdorchik JM (2018). Total hip and knee arthroplasty perioperative pain management: what should be in the cocktail. JBJS Rev.

[10] Wang Q, Tan G, Mohammed A, Zhang Y, Li D, Chen L, Kang P (2021). Adding corticosteroids to periarticular infiltration analgesia improves the short-term analgesic effects after total knee arthroplasty: a prospective, double-blind, randomized controlled trial. Knee Surg Sports Traumatol Arthrosc.

[11] Chung AS, Spangehl MJ (2018). Peripheral nerve blocks vs periarticular injections in total knee arthroplasty. J Arthroplasty.

[12] Huang YS, Lin LC, Huh BK, Sheen MJ, Yeh CC, Wong CS, Wu CT (2007). Epidural clonidine for postoperative pain after total knee arthroplasty: a dose-response study. Anesth Analg.

[13] Kulkarni M, Mallesh M, Wakankar H, Prajapati R, Pandit H (2019). Effect of methylprednisolone in periarticular infiltration for primary total knee arthroplasty on pain and rehabilitation. J Arthroplasty.

[14] Albrecht E, Kirkham KR, Liu SS, Brull R (2013). Peri-operative intravenous administration of magnesium sulphate and postoperative pain: a meta-analysis. Anaesthesia.

[15] Oh TK, Chung SH, Park J, Shin H, Chang CB, Kim TK, Do SH (2019). Effects of perioperative magnesium sulfate administration on postoperative chronic knee pain in patients undergoing total knee arthroplasty: a retrospective evaluation. J Clin Med.

[16] Shin HJ, Kim EY, Na HS, Kim TK, Kim MH, Do SH (2016). Magnesium sulphate attenuates acute postoperative pain and increased pain intensity after surgical injury in staged bilateral total knee arthroplasty: a randomized, double-blinded, placebo-controlled trial. Br J Anaesth.

[17] Zeng J, Chen Q, Yu C, Zhou J, Yang B (2021). The use of magnesium sulfate and peripheral nerve blocks: an updated meta-analysis and systematic review. Clin J Pain.

[18] Li Z, Li Z, Cheng K, Weng X (2021). The efficacy and safety of glucocorticoid on periarticular infiltration analgesia in total knee arthroplasty: a systematic review and meta-analysis of randomized controlled trials. J Arthroplasty.

[19] Hawker GA, Mian S, Kendzerska T, French M (2011). Measures of adult pain: visual Analog Scale for Pain (VAS Pain), Numeric Rating Scale for Pain (NRS Pain), McGill Pain Questionnaire (MPQ), Short-Form McGill Pain Questionnaire (SF-MPQ), Chronic Pain Grade Scale (CPGS), Short Form-36 Bodily Pain Scale (SF-36 BPS), and Measure of Intermittent and Constant Osteoarthritis Pain (ICOAP). Arthritis Care Res (Hoboken).

[20] Xie J, Ma J, Yao H, Yue C, Pei F (2016). Multiple boluses of intravenous tranexamic acid to reduce hidden blood loss after primary total knee arthroplasty without tourniquet: a randomized clinical trial. J Arthroplasty.

[21] Li D, Alqwbani M, Wang Q, Yang Z, Liao R, Kang P (2021). Ultrasound-guided adductor canal block combined with lateral femoral cutaneous nerve block for post-operative analgesia following total knee arthroplasty: a prospective, double-blind, randomized controlled study. Int Orthop.

[22] Wang Y, Feng W, Zang J, Gao H (2020). Effect of patellar denervation on anterior knee pain and knee function in total knee arthroplasty without patellar resurfacing: a meta-analysis of randomized controlled trials. Orthop Surg.

[23] Grosso MJ, Murtaugh T, Lakra A, Brown AR, Maniker RB, Cooper HJ, Macaulay W, Shah RP, Geller JA (2018). Adductor canal block compared with periarticular bupivacaine injection for total knee arthroplasty: a prospective randomized trial. J Bone Jt Surg Am.

[24] Mather LE, Chang DH (2001). Cardiotoxicity with modern local anaesthetics: is there a safer choice?. Drugs.

[25] Hatayama K, Terauchi M, Oshima A, Kakiage H, Ikeda K, Higuchi H (2021). Comparison of intravenous and periarticular administration of corticosteroids in total knee arthroplasty: a prospective, randomized controlled study. J Bone Jt Surg Am.

[26] Tschopp C, Tramèr MR, Schneider A, Zaarour M, Elia N (2018). Benefit and harm of adding epinephrine to a local anesthetic for neuraxial and locoregional anesthesia: a meta-analysis of randomized controlled trials with trial sequential analyses. Anesth Analg.

[27] Tong QJ, Lim YC, Tham HM (2018). Comparing adductor canal block with local infiltration analgesia in total knee arthroplasty: a prospective, blinded and randomized clinical trial. J Clin Anesth.

[28] Laigaard J, Pedersen C, Rønsbo TN, Mathiesen O, Karlsen APH (2021). Minimal clinically important differences in randomised clinical trials on pain management after total hip and knee arthroplasty: a systematic review. Br J Anaesth.

[29] Deckey DG, Verhey JT, Gerhart CRB, Christopher ZK, Spangehl MJ, Clarke HD, Bingham JS (2023). There are considerable inconsistencies among minimum clinically important differences in TKA: a systematic review. Clin Orthop Relat Res..

[30] Jakobsen TL, Kehlet H, Husted H, Petersen J, Bandholm T (2014). Early progressive strength training to enhance recovery after fast-track total knee arthroplasty: a randomized controlled trial. Arthritis Care Res (Hoboken).

[31] Morrell AT, Layon DR, Scott MJ, Kates SL, Golladay GJ, Patel NK (2021). Enhanced recovery after primary total hip and knee arthroplasty: a systematic review. J Bone Jt Surg Am.

[32] Woolf CJ, Thompson SWN (1991). The induction and maintenance of central sensitization is dependent on *N*-methyl-D-aspartic acid receptor activation; implications for the treatment of post-injury pain hypersensitivity states. Pain.

[33] Kreutzwiser D, Tawfic QA (2019). Expanding role of NMDA receptor antagonists in the management of pain. CNS Drugs.

[34] Zhong HY, Zhang WP (2018). Effect of intravenous magnesium sulfate on bupivacaine spinal anesthesia in preeclamptic patients. Biomed Pharmacother.

[35] Woolf CJ, Chong MS (1993). Preemptive analgesia–treating postoperative pain by preventing the establishment of central sensitization. Anesth Analg.

[36] Ugraş AA, Kural C, Kural A, Demirez F, Koldaş M, Cetinus E (2011). Which is more important after total knee arthroplasty: Local inflammatory response or systemic inflammatory response?. Knee.

[37] Jules-Elysee KM, Tseng A, Sculco TP, Baaklini LR, McLawhorn AS, Pickard AJ, Qin W, Cross JR, Su EP, Fields KG, Mayman DJ (2019). Comparison of topical and intravenous tranexamic acid for total knee replacement: a randomized double-blinded controlled study of effects on tranexamic acid levels and thrombogenic and inflammatory marker levels. J Bone Jt Surg Am.

[38] Aryana P, Rajaei S, Bagheri A, Karimi F, Dabbagh A (2014). Acute effect of intravenous administration of magnesium sulfate on serum levels of interleukin-6 and tumor necrosis factor-α in patients undergoing elective coronary bypass graft with cardiopulmonary bypass. Anesth Pain Med.

[39] Bartley EJ, Fillingim RB (2013). Sex differences in pain: a brief review of clinical and experimental findings. Br J Anaesth.

